# The effect of menu labeling with calories and exercise equivalents on food selection and consumption

**DOI:** 10.1186/s40608-014-0021-5

**Published:** 2014-09-24

**Authors:** Charles Platkin, Ming-Chin Yeh, Kimberly Hirsch, Ellen Weiss Wiewel, Chang-Yun Lin, Ho-Jui Tung, Victoria H Castellanos

**Affiliations:** City University of New York School of Public Health, Hunter College, Nutrition and Food Science Program, Hunter College, Silberman Bldg, 2180 Third Avenue, New York, NY 10035 USA; CUNY School of Public Health, Graduate Center, New York, USA; Department of Applied Mathematics and Institute of Statistics, National Chung Hsing University, Taichung City, Taiwan; Department of Healthcare Administration, Asia University, Taichung City, Taiwan; The University of Scranton, Scranton, PA USA

**Keywords:** Menu labeling, Nutrition labeling, Exercise equivalents, Point-of-purchase, Fast food, Obesity

## Abstract

**Background:**

Better techniques are needed to help consumers make lower calorie food choices. This pilot study examined the effect of menu labeling with caloric information and exercise equivalents (EE) on food selection. Participants, 62 females, ages 18-34, recruited for this study, ordered a fast food meal with menus that contained the names of the food (Lunch 1 (L1), control meal). One week later (Lunch 2 (L2), experiment meal), participants ordered a meal from one of three menus with the same items as the previous week: no calorie information, calorie information only, or calorie information and EE.

**Results:**

There were no absolute differences between groups in calories ordered from L1 to L2. However, it is noteworthy that calorie only and calorie plus exercise equivalents ordered about 16% (206 kcal) and 14% (162 kcal) fewer calories from Lunch 1 to Lunch 2, respectively; whereas, the no information group ordered only 2% (25 kcal) fewer.

**Conclusions:**

Menu labeling alone may be insufficient to reduce calories; however, further research is needed in finding the most effective ways of presenting the menu labels for general public.

## Background

Point-of-purchase menu labeling, particularly at fast food restaurants, has been of special interest in the fight against obesity. As fast food consumption has been correlated with obesity and other negative health outcomes, these food outlets are being targeted for change. In 2010, the US federal health care reform bill was signed into law and includes a requirement that restaurant chains with at least 20 outlets nationwide post calorie labels on menu boards [1]. It has been suggested that knowledge of the calories contained in foods is essential to choosing and consuming an energy-balanced diet [2,3]. While consumer polls show a desire for calorie information at the point-of-purchase in restaurants [4], research on the actual effects has shown mixed results [5-7].

One possible reason for inconsistent effectiveness may be the lack of understanding of the value of a calorie or the lack of a reference amount for a calorie [8,9]. Exercise equivalents have been discussed by nutrition experts as a potential method to inform consumers about calorie values [10]. Exercise equivalents are defined as the amount of time doing particular physical activities that would be needed to burn off calories in foods. For example, burning off a 300-calorie hamburger would require about 75 minutes of walking, after expending the calories needed for daily subsistence. Exercise equivalents could potentially simplify food and/or restaurant nutrition labels, increase understanding of calories and of energy imbalance, and facilitate a decrease in overall energy intake [11].

Literature exploring the use of exercise equivalents is limited. A new study by Dowray et al., explored the potential effect of exercise equivalents on menu labels. This study was a web-based survey, and asked participants to “imagine they are in a fast food restaurant”, and order a meal from an online menu. Participants were randomly assigned to see one of four menus (calories only, calories and number of minutes to walk to burn off that amount of calories, calories and number of miles to walk off that amount of calories, or no information). The results from this study are significant; calories were significantly different based on menu type (p = 0.02), with the calories and exercise equivalents in mileage group ordering significantly less calories than the other three groups (p = 0.0007). Additionally, 82% of their participants reported a preference for exercise equivalents on menu labeling [12]. This study shows a positive impact in using exercise equivalents to aid in the understanding of a calorie, and potential to help consumers order lower calorie food items. These findings are consistent with an earlier study, which showed that exercise equivalents helped reduce purchases of sugar-sweetened beverages among low-income black adolescents [11].

A study by Fitch et al. assessed the influence of calorie information versus exercise equivalents on food selection amongst adolescents and adults who ate at fast food restaurants regularly [13]. This study indicated that calorie labels were preferred to exercise equivalents overall (71%), and some cited the latter as demotivating [14]; however, the Fitch study has limitations. They tested the impression of exercise equivalents rather than their actual effect, examined exercise equivalents as an alternative to, rather than addition to calories, and had a predominantly white sample population, many of whom were not overweight or obese. They also found that exercise equivalents had a more favorable impression among non-whites than whites, and, along with another study [14], among younger persons.

The current study aimed to test the actual effect of exercise equivalents on fast food point-of-purchase behaviors. Research has shown that non-white, overweight and obese individuals are more likely to consume fast food and thus be at increased risk of negative health outcomes [15]. For this reason, we recruited young, predominantly non-white overweight and obese women for our study and presented them with exercise equivalents alongside calories at the point-of-choice. We compared the effect of the exercise equivalents with the provision of simple caloric information or no information at all. Additionally, we sought to evaluate the impact of restrained eating on point of purchase and consumption behaviors.

The current study was a pilot. While the researchers acknowledge the small sample size as a limitation, the goal of the study was to test a new design: the potential use of exercise equivalents for public health outreach, with hopes that other researchers can utilize for future studies on this topic. Thinking of new ways to promote healthy behaviors at the point-of-purchase is important for nutrition researchers, educators, and policymakers. We believe the novelty of our experimental design, with emphasis on using exercise equivalents as a nutrition intervention for at risk individuals, furthers thought on point-of-purchase interventions.

## Methods

### Study overview

A three-group repeated-measures experimental study was conducted to determine whether providing information about calories and exercise equivalents at the point-of-choice for a fast food meal would decrease calories ordered or consumed among overweight and obese 18-34-year-old women at a public university in southern Florida in 2009, and to investigate any correlation with consumption with prior dieting history, qualified as dietary restraint in this study. All participants were asked to participate in two sessions during a two-week period. The Florida International University Institutional Review Board approved this study. All persons gave their informed consent prior to inclusion in the study.

### Study participants

A total of 62 overweight or obese female participants were recruited on a south Florida college campus. Telephone and in-person screening determined whether participants met the inclusion criteria: female, age 18-34 years old, BMI at least 25 and less than 40, as calculated from researcher-measured height and weight, ate fast food at least “occasionally”, and able to read and speak English. Participants were also screened at this time for dietary restraint for randomization into the three study groups. Persons were excluded for dieting in the last three months; requiring a special diet such as vegetarian, kosher, or accommodating a food allergy or health condition; being pregnant or giving birth in last year; having a chronic disease such as heart disease or diabetes; having current self-reported depression, self-reported alcohol or drug abuse, or eating disorder; being a health major; not typically eating lunch; and participating in a previous food-related study. Exclusion criteria were set to ensure participants were healthy and able to partake in a food-related study and to help avoid any bias gained from previous food-related studies. In order to help further blind participants to the menu manipulation aspect of the study, participants were told that the purpose of the study was to “better understand fast food meal choices”.

### Experimental design

Participants attended two meal sessions, Lunch 1 and one week later Lunch 2. The food choices were from a fast food restaurant located on the university campus. The restaurant is part of a national chain specializing in hamburgers and French fries. The foods were in their original portion-controlled wrappers or packaging, which allowed the researcher to easily record choices made by participants. The study took place in a controlled setting within the university, at a private conference room in the University’s Graham Center nearby the student union where the students normally eat. Incentives for the participants included $5 for completion of the screening questions, the two free lunches, and a $20 gift card at each lunch.

At the start of each Lunch, participants were given a menu. The paper menus were in a similar format to menu boards at fast food restaurants. The food items were those available for lunch at Burger King on the dates of the experiment. The participants were able to choose entrées (e.g. Hamburger, Whopper, TenderGrill, BK Veggie Burger or TenderGrill), a garden salad, side dishes (i.e., fries, onion rings), condiments (ketchup, mayonnaise, fat free ranch dressing, or honey mustard dressing) and a drink (i.e., water, Coca-Cola, diet Coca-Cola, or apple juice). The observer recorded the quantity of the food ordered and eaten by using a digital food scale, weighing the remaining portions and using a measuring cup for the liquids. The researcher ensured that all participants had finished eating and had left the study site prior to weighing and measuring left-over foods and drinks. Calories consumed were derived by taking food waste and weighing on a digital scale and calculating total calories eaten by the following formula: Total Calories For Food Item Chosen–Food Waste = Calories Consumed.

Lunches were served from 11:30 a.m. until 3:00 p.m., and participants made appointments at 30-minute increments. All participants were told in advance that they would not be able to leave the study site with any leftover food, to limit the possibility that participants would order more food than they intended to consume. During Lunch 1, participants were given a menu, similar in format to menu boards at fast food restaurants. Participants were able to order any foods and beverages from the menu, which listed only names of items, no calories or exercise equivalents.

The experimental manipulation took place one week later at Lunch 2. All participants were randomly assigned to one of three groups. Each group received different information on their menus: no information on calorie or exercise equivalents, calories only, or calories and exercise equivalents. Column headers for the exercise equivalents and calories described the numbers (“minutes to burn off food in walking”, “calories”), as did labels after each values. The exercise equivalence of calories was calculated based on an intensity level of 3.3 METs for walking at the moderate pace of 3.0 mph on a firm surface [16], and a body weight of 160 pounds.

### Data collection

At the research table, participants completed standardized questions on the following demographic information: age, marital status, education, income, race, religion, and whether the participant was a smoker. Body Mass Index was assessed by the investigators at the research table using a standardized height and weight measurement procedure as outlined in Third National Health and Nutrition Examination Survey (NHANES III) Anthropometric Procedures Manual [15].

Dietary restraint was determined using the TFEQ [17]. Scores on the TFEQ restraint sub-scale range from 0 to 21, with restrained eaters defined as those who have a score of 13 or above. Participants were blocked by restraint in order to test whether unrestrained and restrained eaters responded differently, since restraint has been shown to influence food choice and the reading of nutrition labels [18,19], and were then randomly assigned to one of three study groups.

### Statistical analysis

ANOVA and chi-square tests were conducted to compare demographic information by study group. Both the foods ordered and the foods consumed were analyzed. Within each study group, a paired t-test was conducted to test for the change in calories ordered or consumed from Lunch 1 to Lunch 2. The subsequent change by study group was calculated as the mean (plus or minus the standard error) of the changes from Lunch 1 to Lunch 2 for each of the group’s individual members.

Proportionate change for calories order and calories consumption from Lunch 1 to Lunch 2 were calculated as the mean (plus or minus the standard error) of the proportionate changes of each of the group’s individual members. The t-tests were used to examine whether the proportionate changes are significant.

Analysis of covariance (ANCOVA) was conducted using General Linear Model in SPSS 17.0 statistical software (SPSS Inc., 2008) to test for differences between study groups in difference from Lunch 1 to Lunch 2 in calories ordered or consumed. The effect size (partial eta squared) and observed power (using alpha = 0.05) are also calculated using SPSS. Two general linear models were created, both controlling for age, BMI, and dietary restraint, and with study group as the fixed factor. For model 1, the response variable was the difference from Lunch 1 to Lunch 2 in total calories ordered, and an additional covariate was calories ordered in Lunch 1 (control meal). For model 2, the response variable was the difference from Lunch 1 to Lunch 2 in total calories consumed, and an additional covariate was calories consumed in Lunch 1. Both models assumed that the response variables were continuous, residuals were normally distributed, and the subjects were independent. Total number of items ordered was also compared by study group.

## Results

Study participants had a mean age of 21.9 ± 3.03 years and BMI of 28.4 ± 3.10. Seventy-three percent (n = 45) were black or Hispanic and 63% (n = 39) were unrestrained eaters (Table 1). Demographic information was comparable across study groups (all *p* values > 0.05) (Table 1). All study groups decreased the number of calories ordered from Lunch 1 to Lunch 2 (Table 2). While the current study is under-powered to ascertain statistical non-significance or significance, calorie only and calorie plus exercise equivalents ordered about 16% (206 kcal) and 14% (162 kcal) fewer calories from Lunch 1 to Lunch 2, respectively; whereas, the no information group ordered only 2% (25 kcal) fewer (Table 2).

**Table 1:** **Participant characteristics by study group, in a group of overweight or obese women**

	**Study group for menu type at Lunch 2 (experiment meal)**						
	**No calorie or exercise equivalent information**		**Calories only**		**Calories and exercise equivalents**		**p** ^**1**^
Total	N	%	N	%	N	%	
	22	100%	20	100%	20	100%	
Age (years; mean, SD)	21.9 ± 3.5		21.6 ± 2.3		22.2 ± 3.2		0.82
Weight (pounds; mean, SD)	167.9 ± 26.5		171.2 ± 26.6		165.6 ± 25.8		0.79
BMI (kg/cm^2^; mean, SD)	27.9 ± 3.1		28.7 ± 3.0		28.7 ± 3.3		0.64
Race/Ethnicity (N)							0.90
Hispanic/Latino	8	36%	10	50%	10	50%	
Black/African American	7	32%	5	25%	5	25%	
Other	7	32%	5	25%	5	25%	
Dietary restraint^2^							0.66
Restrained	7	32%	7	35%	9	45%	
Unrestrained	15	68%	13	65%	11	55%	

^1^Using ANOVA for age, weight and BMI, and Chi-square test for dietary restraint and race/ethnicity.

^2^Classified using restraint subscale of TFEQ; score of <13 indicates restrained eater, > = 13 indicates unrestrained eater.

**Table 2:** **Calories ordered and consumed (mean ± SE) by meal and study groups**

	**Model 1: Calories ordered (mean ± SE) by meal** ^**1**^						
**Study group**	**n**	**Lunch 1** ^**3**^	**Lunch 2**	**Difference**	**Proportionate change (%)** ^**4**^	**p**	**Cohen’s d**
No calorie or exercise equivalent information	22	1,201.4 ± 100.0	1,176.1 ± 99.5	-25.2 ± 95.2	9.3 ± 11.6	0.43	0.17
Calories only	20	1,282.8 ± 89.7	1,077.0 ± 114.0	-205.8 ± 110.6	-14.4 ± 7.3	0.06	0.45
Calories and exercise equivalents	20	1,162.8 ± 141.1	1,000.5 ± 98.2	-162.3 ± 132.5	1.6 ± 13.3	0.90	0.02
	**Model 2: Calories consumed (mean ± SE) by meal** ^**2**^						
**Study Group**	**n**	**Lunch 1** ^**3**^	**Lunch 2**	**Difference**	**Proportionate change (%)** ^**5**^	**p**	**Cohen’s d**
No calorie or exercise equivalent information	22	986.6 ± 84.1	995.4 ± 91.5	8.8 ± 83.9	10.7 ± 11.6	0.36	0.2
Calories only	20	1,059.6 ± 72.7	898.8 ± 87.6	-160.7 ± 106.3	-9.3 ± 10.7	0.39	0.2
Calories and exercise equivalents	20	840.9 ± 88.6	841.3 ± 82.0	0.5 ± 76.9	11.9 ± 13.7	0.40	0.2

^1^ANCOVA p-value = 0.43, controlling for age, BMI, race, dietary restraint, and calories ordered at Lunch 1; partial eta squared = 0.03, observed power = 0.19.

^2^ANCOVA p-value = 0.31, controlling for age, BMI, race, dietary restraint, and calories consumed at Lunch 1; Partial eta squared = 0.04, observed power = 0.25.

^3^All persons received menus with no calorie or exercise equivalent information at Lunch 1.

^4^Overall mean ± SE of individual proportionate changes, each calculated as (calories ordered in Lunch 2-calories ordered in Lunch 1)/calories ordered in Lunch 1.

^5^Overall mean ± SE of individual proportionate changes, each calculated as (calories consumed in Lunch 2-calories consumed in Lunch 1)/calories consumed in Lunch 1.

In all study groups, both restrained and unrestrained eaters had an average decrease in number of calories ordered from Lunch 1 to Lunch 2, with the exception of restrained eaters in the group receiving no calorie or exercise equivalent information at Lunch 2. The greatest proportionate decrease in calories ordered was seen in restrained eaters in the calories-only group (24.7% decrease; *p* = 0.05). Unrestrained eaters in the calories and exercise equivalents group ordered an average of 275 fewer calories at Lunch 2 compared with Lunch 1, with a proportionate decrease of 14.0%, although this was not statistically significant (*p* = 0.24). Unrestrained eaters in the calories and exercise equivalents group had greater absolute and proportionate decreases in calories ordered from Lunch 1 to Lunch 2 than unrestrained eaters in the other two study groups. Additional analyses examining number of items ordered revealed no significant differences by study group (data not shown).

During the exit questionnaire, 57 participants (92%) said they believed that a combination of calories and exercise equivalents would influence the foods they ordered at a fast food restaurant.

## Discussion

Consumption of fast foods is common in the US [20]. To reduce negative effects and mitigate public health disparities in food environments, interventions may be especially critical in populations of persons who eat at fast food restaurants [21]. Calorie information presented at point-of-purchase is a relatively new concept nationwide; however, research has shown mixed results at the point of purchase. There is a potential for exercise equivalents as a supplemental guide to novice calorie counters and those unaware of the negative health implications in consuming fast food.

While the current study was underpowered, we believe the novelty of the design of the experiment, the emphasis on utilizing exercise equivalents for a potential nutrition intervention for at risk individuals, will heighten awareness for future researchers on the need for further investigating point of purchase interventions, specifically exercise equivalents. There have been several real-world studies that have shown an impact on calories ordered using sales data. For example, using a randomization design, Roberto et al. reported that calorie information on restaurant menu did reduce the total amount of calories people ordered and consumed [22]. Another quasi-experimental design study [23] examined the sales data before and after provision of point-of-selection nutrition labels found that the nutrition labels reduced average energy content of entrée purchased without reducing overall sales. Additionally, using data from Starbucks, Bollinger et al. found that mandatory calorie posting in chain restaurants resulted in 6% decrease in calories per transaction [24]. Dumanovsky et al. conducted a cross-sectional survey and assessed consumer purchases in 2007, before caloric information was mandated by chain restaurants, and again in 2009, after the menu labeling legislation was passed. Although they did not find an overall change in calories consumed, they did observe a significant decrease in the calories consumed at specific chain restaurants including McDonald’s, Au Bon Pain and KFC [25].

With the rollout of the new law mandating fast food restaurants list caloric value for all menu items pending, understanding the potential implications is important. Calorie information at the point-of-purchase for restaurants has been required by law for chain restaurants in New York City since 2008 [26], in California, Oregon and Maine since 2009 [27] and has also been adopted in many other cities and counties [28].

A recent study by Krieger et al. is one of the first to investigate the effect of the nationwide menu labeling bill. This cross-sectional study surveyed fast food patrons both before the menu label regulation was implemented, and again 18 months later, post-regulation. Interestingly, they found a significant decrease in calories ordered in coffee and taco establishments, but not in burger and sandwich shops; and a decrease in calories ordered by women, but not men [29].

The effectiveness of nutrition labels on point-of-choice food purchasing has provided mixed results. Similar to the present study, prior studies that have looked at point-of-purchase at fast food restaurants and the other at nutrition labels have also failed to show statistical significance [30,31]. This study provided a real-world setting was created to measure actual point-of-purchase behavior [32-34]. The unique strength of this study is that the study design provides a potential alternative or addition to the soon-to-be implemented national menu labeling law as a public health intervention. This study illustrates a novel design to test the effectiveness of adding exercise equivalents to provide a frame of reference for consumers. Using exercise equivalents on food labels and food served away from home could provide consumers with a context for the term, “calorie”, [11] and, thus, contribute to the understanding of the nutrition labels for better food choice and selection.

Presentation of caloric information of fast food translated into exercise equivalents did not have a statistically significant impact on the food choices of overweight and obese women who were restrained eaters or unrestrained eaters. However, unrestrained eaters presented with calorie information and exercise equivalents combined had a larger decrease in calories ordered compared to those with calorie information only and for those with no information. The impact of calorie information with exercise equivalents on unrestrained eaters should be further examined as unrestrained eaters generally do not deliberately attempt to limit their food intake [17].

There are several limitations in this exploratory study. The small study sample size is a major limitation of this study. Additionally, the study was limited to female college students thus limiting its generalizability. Another limitation is that individuals were getting food at no cost, which might have influenced the total number of food items, and hence amount of calories chosen [30]. The average calories for the foods chosen for Lunch 1 and Lunch 2 were 1215.16 and 1087.50, respectively. Dumanovsky and colleagues (2009) established baseline data on mean calorie intake at Burger King of 926.2. Participants in the current study chose approximately 225 more calories per meal on average than participants in Dumanovsky study, perhaps because the food was free [31]. This study did not collect pre- and post-intervention food diaries. It is possible that participants who chose lower calorie foods during the intervention may have increased their intake later in the day to compensate.

Additionally, there is a potential limitation in using exercise equivalents, or calories alone, to promote lower calorie food choices as opposed to nutritionally dense options. Lower calorie foods do not necessarily make a food nutritionally “better” than another. Despite this, although nutritionally and calorically dense foods such as tree nuts, avocado, and fatty fish are touted as an important part of a healthful diet, they are not commonly offered at fast food restaurants. As such, exercise equivalents and calories listed can potentially provide meaningful reference points for fast-food patrons [32].

## Conclusions

The current study presented an intervention designed to improve the effectiveness of calorie information on point-of-choice. The concept of menu labeling, exercise equivalents and other point-of-purchase messages are a potentially useful way to reach consumers at the point of their food decision. This research, combined with previous studies, suggest that in addition to calorie labeling information, there is a need for further research of point-of-purchase interventions [34] to find the most effective ways of presenting the menu labels for general public. Although this study was not powered to see statistical differences, the concept that behaviors may differ based on calorie and exercise information should be further explored in a larger study.
